# Quercetin protects porcine oocytes from *in vitro* aging by reducing oxidative stress and maintaining the mitochondrial functions

**DOI:** 10.3389/fcell.2022.915898

**Published:** 2022-10-05

**Authors:** Yafei Jiao, Yixian Wang, Tiantuan Jiang, Keying Wen, Peiqing Cong, Yaosheng Chen, Zuyong He

**Affiliations:** State Key Laboratory of Biocontrol, School of Life Sciences, Sun Yat-sen University, Guangzhou, China

**Keywords:** quercetin, oxidative stress, mitochondrial function, porcine oocytes, *in vitro* aging

## Abstract

Quercetin (QUE) is a component of the flavonoid family that shows various therapeutic properties, such as antioxidant effects. However, whether QUE affects porcine oocyte *in vitro* aging has not yet been investigated. Therefore, in this study, we applied various doses of QUE to freshly isolated porcine oocytes and found that 10 µM QUE improved the oocyte maturation rate *in vitro*, as reflected by the increased degree of cumulus cell expansion and first polar body extrusion. More importantly, we found that QUE reduced *in vitro* aging and improved the maturity level of porcine oocytes after another 24 h of culturing, accompanied by the upregulated expression levels of bone morphogenetic protein 15, growth differentiation factor 9, Moloney sarcoma oncogene, and cyclin-dependent kinase 2. In addition, we found that QUE treatment significantly reduced the intracellular reactive oxygen species levels, apoptosis, and autophagy and upregulated the expression levels of superoxide dismutase 2 and catalase in aged porcine oocytes. In addition, QUE restored impaired mitochondrial membrane potential and spindle assembly in aged porcine oocytes. Our findings demonstrate that QUE can protect porcine oocytes from *in vitro* aging by reducing oxidative stress and maintaining mitochondrial function.

## Introduction

*In vitro* maturation (IVM) of mammalian oocytes is one of the most important processes for subsequent early embryonic development and implantation (Leoni et al., 2009). The IVM rate of porcine oocytes (approximately 77%) (Nie et al., 2020) is lower than that of other species, such as humans (approximately 94%) (Srivastava et al., 2006; Sudha et al., 2006) and cattle (approximately 86%) (Hoshi 2003). The IVM of porcine oocytes involves complex metabolic activity; hence, strategies for disrupting certain metabolic pathways may improve the IVM rate (Luo et al., 2020; Nie et al., 2020). Reactive oxygen species (ROS) are common metabolites produced during IVM of oocytes. Physiological levels of ROS are necessary for normal development of oocytes, and ROS act as signaling molecules and regulate the cell development cycle and apoptosis (Guemra et al., 2013). However, high levels of ROS can be generated during IVM because of excessive *in vitro* manipulation of oocytes and hyperoxia stress, which is a negative factor affecting the IVM rate of oocytes (Pu et al., 2014). One of the negative effects of high ROS levels on oocyte IVM is the induction of oocyte apoptosis (Kang et al., 2013).

In recent years, an increasing number of studies have shown that natural antioxidants can delay oocyte *in vitro* aging. Putrescine is a common biogenic amine. Supplementation of the M2 culture medium with putrescine can improve the quality of aged mouse oocytes by reducing the levels of ROS and increasing the expression levels of pyruvate dehydrogenase kinase 4 (Xu et al., 2018). Resveratrol can alleviate post-ovulatory aging of mouse oocytes by maintaining the cytoskeleton morphology, preventing early apoptosis, and protecting the loss of methylation of H3K9me2, resulting in improved binding of aged oocytes to sperms and enhanced embryo developmental potential (Sun et al., 2019). Supplementation of allicin in TCM-199 mature medium alleviates apoptosis and autophagy in aged porcine oocytes and increases the development rate of parthenogenetic porcine embryos to the blastocyst stage (Park et al., 2019). Supplementation with porcine zygote medium-3 can improve the parthenogenetic embryonic development of aged porcine oocytes by reducing the level of DNA methylation and abnormal histone methylation (Nie et al., 2018). QUE is a powerful antioxidant and free radical scavenger owing to its phenolic ring structure (Hamza, El-Shenawy, and Ismail 2015). QUE reacts with a free radical by donating a proton and becomes a radical itself because of the formation of an unpaired electron. However, this unpaired electron is delocalized by resonance, making the QUE radical non-reactive because of its low energy (Mariani et al., 2008). QUE can reduce mouse oocyte aging by modulating the expression of sirtuins and inhibiting oocyte quality deterioration (Wang et al., 2017). Furthermore, QUE has positive effects on the IVM of bovine oocytes and early embryonic development (Sovernigo et al., 2017). QUE supplementation can reduce intracellular ROS levels to promote porcine oocyte maturation and parthenogenetic embryo development to the blastocyst stage (Guemra et al., 2013).

To date, whether QUE can improve the quality of aged porcine oocytes has not been reported. Therefore, we investigated whether QUE could prevent the *in vitro* aging of porcine oocytes*.* Moreover, the effects of QUE on the IVM of porcine oocytes, morphology of aged porcine oocytes, ROS production, mitochondrial function, spindle organization, early apoptosis, and autophagy in aged porcine oocytes were analyzed.

## Materials and methods

### Chemicals and reagents

Fetal bovine serum (FBS) was purchased from Hyclone (Logan, United, United States). The Cells-to-cDNA™ II Kit was purchased from Ambion (Austin, TX, United States). All other experimental chemicals and reagents were obtained from Sigma–Aldrich (St. Louis, MO, United States).

### Isolation and *in vitro* culture of porcine oocytes

Ovaries were collected from a local slaughterhouse in Guangzhou. The mesovarium was removed and the ovaries were rinsed with saline and placed in a physiological saline solution bath at 38 ° C. Porcine cumulus–oocyte complexes (COCs) were aspirated from 3–8 mm ovarian follicles using a 10-ml syringe with an 18-gauge disposable needle. Then, 20–30 COCs were cultured in 150 μl of IVM medium containing TCM-199, 10% (v/v) FBS, 10% (v/v) porcine follicular fluid (PFF), 10 IU/ml follicle-stimulating hormone (FSH), 10 IU/ml luteinizing hormone (LH), and 50 IU/ml penicillin and streptomycin (Cai et al., 2021); covered with mineral oil; and cultured for 42–44 h at 38.5° C in a 5% CO_2_ incubator with 95% air. COC expansion was characterized by three grades. Grade 1: no significant morphological change was observed compared with freshly recovered COCs; Grade 2: COCs expanded slightly, but cell–to–cell contact was still relatively tight, with clustered cells still observed; Grade 3: COCs were extensively expanded, and cumulus cells were homogeneously spread (Kobayashi, Yamashita, and Hoshi 1994).

### *In vitro* aging of porcine oocytes and QUE treatment

After the COCs were cultured for 42–44 h *in vitro* with a mature medium, the CCs were removed from the COCs with 0.1% hyaluronidase, which was diluted in TL-PVA-HEPES to harvest naked oocytes. Oocytes that had extruded the first polar body (PB) were cultured in a mature medium supplemented with (QUE-Aged) or without (Aged) QUE for another 24 h at 38.5°C in a 5% CO_2_ incubator with 95% air. QUE (Q4951; Sigma, United States) was dissolved in 5 μl dimethyl sulfoxide (DMSO) and diluted to final concentrations of 5, 10, and 20 µM in a culture medium, according to a previously reported study (Cao et al., 2020). DMSO concentration was maintained below 0.05%. Oocytes with fragmented morphology, disjointed ooplasm, and parthenogenetic activation were judged as aged oocytes, based on previous studies (Kikuchi et al., 1995; Gable and Woods 2001). The aging rate was calculated as the percentage of both fragmented and parthenogenetic activated oocytes.

### ROS level measurement

The 2′, 7′-dichlorodihydrofluorescein diacetate (DCFH-DA) fluorescent probe (Beyotime Biotechnology) was diluted with phosphate-buffered saline (PBS) at 1:1,000 to achieve a final concentration of 10 µM. DCFH-DA itself does not emit fluorescence; however, when DCFH is oxidized by intracellular ROS, it becomes fluorescent DCF. Oocytes were incubated with 10 µM DCFH-DA fluorescent probe solution for 40 min at 38 ° C in dark. The oocytes were then rinsed three times with PBS-PVA. Images were captured using a microscope (Eclipse Ti2-E, Nikon, Japan) with the same scanning settings for all groups. The oocyte fluorescence signal was analyzed using ImageJ software (National Institutes of Health, Bethesda, MD, United States).

### ATP level measurement

The ATP assay kit (Beyotime Biotechnology, Shanghai, China) was used to measure ATP levels in porcine oocytes. Briefly, thirty oocytes were lysed with 20 μl of lysis buffer, followed by centrifugation at 400 × g for 5 min at 25 C to collect the supernatant. The ATP level was determined by mixing 20 μl of the supernatant with 100 μl of the luciferase working solution. The fluorescent signal was generated by catalysis of luciferin by firefly luciferase powered by ATP. The intensity of the fluorescent signal was linearly related to ATP concentration, which was measured using a microplate luminometer (BioTek, United States).

### Mitochondrial membrane potential measurement

The MMP in porcine oocytes was measured using an MMP assay kit (Beyotime Biotechnology, Shanghai, China), according to the manufacturer’s instructions. Briefly, 50 μl JC-1 (200 ×) was mixed with 8 ml DEPC water through a strong vortex, and then 2 ml JC-1 staining buffer (5×) was added to form the JC-1 staining working solution. A total of eight oocytes from each group were added into the working solution stained at 38 ° C for 30 min. Carbonyl cyanide m-chlorophenylhydrazone (CCCP) was used as the positive control. CCCP is a reversible mitochondrial uncoupler. Treatment of porcine oocytes with 10 μM CCCP for 20 min resulted in the loss of mitochondrial membrane potential, and JC-1 stained green. Images were captured using a microscope (Eclipse Ti2-E, Nikon, Japan) with the same scan settings across multiple groups. Oocyte fluorescence signal was analyzed using ImageJ software.

### Oocyte early apoptosis assay

The occurrence of early apoptosis in porcine oocytes was tested using an Annexin V-FITC/PI apoptosis measurement kit (Vazyme, China) according to the manufacturer’s instructions. Briefly, 5 μl of annexin V-FITC and 400 μl of 1× binding buffer were gently mixed to form the working solution. A total of eight oocytes were placed in the annexin V-FITC working solution and incubated at 38° C for 30 min. Annexin V is a Ca^2+^-dependent phospholipid-binding protein that has a high affinity for phosphatidylserine, whereas, in normal cells, phosphatidylserine is only distributed on the inner side of the lipid bilayer of the cell membrane, and in the early stage of apoptosis, phosphatidylserine in the cell membrane is turned from the inside to the outside of the lipid membrane. Therefore, annexin V is recognized as a sensitive indicator of early apoptosis in cells. Images were captured using a microscope (Eclipse Ti2-E, Nikon, Japan) with the same scan settings across multiple groups. The oocyte fluorescence signal was analyzed using ImageJ software.

### Quantitative reverse transcription PCR

The oocytes were collected into PCR tubes containing 8 μL cell–to–cDNA™ II lysis buffer. Next, oocyte RNA was extracted and reverse-transcribed to cDNA using the SuperScript ™ II Reverse Transcriptase Kit (Invitrogen, Carlsbad, CA, United States) following the manufacturer’s instructions. Briefly, the following is the 20-µl mixture of reverse transcription system containing 1 µL of DNase I, 1.3 µl of DNase I buffer, 1 µl of EDTA, 1 µl of dNTP, 2 µl of random primer, 4 µl of 5x First-Strand Buffer, 0.5 µl of RNase inhibitor, 2 µl of DTT, and 0.3 µl of FS RT (reverse transcriptase). Next, quantitative PCR was carried out to detect cDNA, and all primers used for mRNAs (Supplementary Table S1) were designed using Oligo 7.0 and compounded by Sangon Biotech (Sangon Biotech, Shanghai, China). The levels of gene transcripts were quantified using 2× Taq Pro Universal SYBR qPCR Master Mix (Vazyme, Nanjing, China), and fluorescence signals were acquired using a fluorescence proportion PCR instrument (Applied Biosystems, QuantStudio 7 Flex, Singapore). The sample was analyzed three times, and the relative gene expression was computed using the 2^−ΔΔCt^ method with glyceraldehyde-3-phosphate dehydrogenase (*GAPDH*) as the housekeeping gene.

### Immunofluorescent staining

Oocytes were fixed with 4% PFA at 4 ° C overnight and then rinsed thrice with a blocking solution (PBS containing 3% bovine serum albumin [BSA] and 7.5% glycine). The oocytes were permeabilized with 1% Triton X-100 in PBS for 30 min at 25 ° C and blocked with 1% BSA at 25 ° C. The oocytes were then washed thrice with 1% Triton X-100 and 3% BSA in PBS. Next, oocytes were incubated with an anti-α-tubulin FITC primary antibody at an attenuation of 1:200 and an anti-LC3B antibody at an attenuation of 1:200 overnight at 4 ° C (Supplementary Table S2). After that, the oocytes were washed with PBS-PVA three times and incubated with a secondary antibody (Supplementary Table S2) for 1.5 h at 25 ° C. For spindle structure analysis, oocytes were counter-stained with 10 μg/ml 2-(4-amidinophenyl)-6-indolecarbamidine dihydrochloride for 15 min and then washed three times with PBS-PVA. Finally, fluorescent images were captured using a microscopy system (LAS—Leica TCS-SP5, Germany) using the same scanning settings across different samples, and the fluorescent signal of the oocytes was analyzed using ImageJ software.

### Western blotting assay

Two hundred oocytes were lysed with 120 μl radioimmunoprecipitation assay lysis buffer (Beyotime Biotechnology, Shanghai, China) in an EP tube. The protein concentration was determined using a protein assay kit (Beyotime Biotechnology, Shanghai, China). Next, 30 μl of 5× loading solution per lysed sample was added. A total of 30 μg of protein was transferred to a nitrocellulose membrane using a semi-drying transfer system (Bio-Rad, Hercules, CA, United States). After blocking for 1.5 h with 3% BSA in PBS, the membrane was incubated with a primary antibody (Supplementary Table S2) overnight at 4 °C. Following three 10-min washes in Tris-buffered saline containing 0.1% (v/v) Tween-20, the membrane was incubated with a secondary antibody in Supplementary Table S2 for 1 h at 25 °C. The membrane was washed with Tris-buffered saline containing 0.1% (v/v) Tween-20 and then covered with a 1:1 mixture of enhancer solutions A and B (Millipore, Darmstadt, Germany) for 3 min. Protein bands were visualized using an imaging system (Bio-Rad, Hercules, CA, United States), and the intensity of the bands was analyzed using ImageJ software. Protein levels were standardized by comparison with GAPDH.

### Statistical analysis

All experiments were performed in triplicate. Statistical analysis was performed using SPSS software version 17.0, and quantitative data were presented as the mean ± standard error of the mean. Statistical differences between two groups were analyzed using the *t*-test, and Duncan’s test was used for analysis of variance in more than two groups. *p*-values < 0.05 were considered to be statistically significant, and *p*-values < 0.01 were considered to be extremely statistically significant.

## Results

### QUE supplementation promotes porcine COC expansion and increases porcine oocyte maturation rate

**FIGURE 1:**
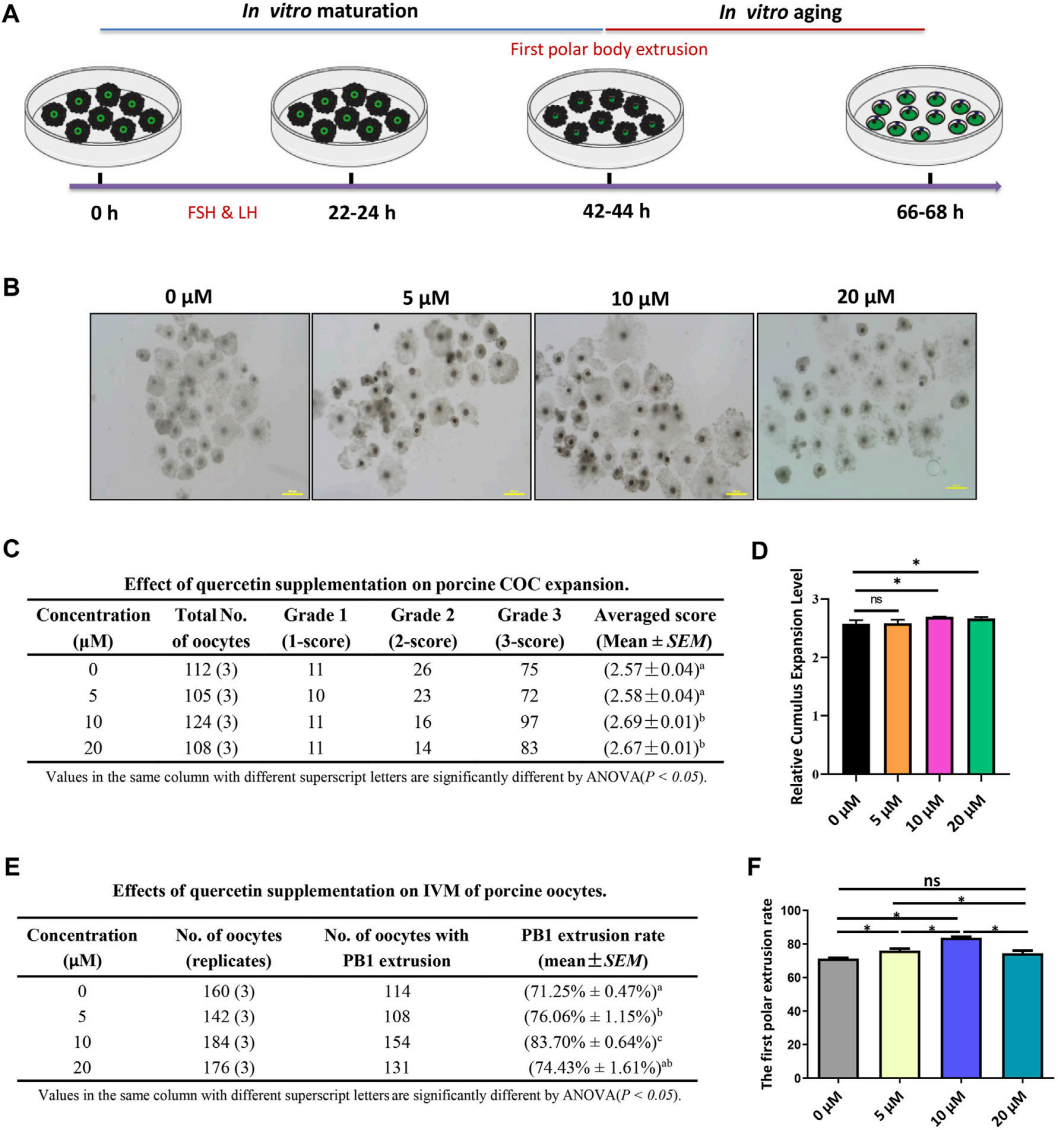
Quercetin supplementation improves the *in vitro* maturation (IVM) of porcine oocytes. **(A)** Diagram of IVM and aging of porcine oocytes. **(B)** Representative images of 0, 5, 10, and 20 μM QUE on porcine cumulus oocyte complex (COC) expansion (n = 449). **(C)** Effect of QUE on porcine COC expansion. Analysis of variance (ANOVA) of different superscript letter values in the same column is significantly different (*p* < 0.05) (n = 662). **(D)** Statistical analysis of the effects of different concentrations of QUE on porcine COC expansion. The symbol * indicates significant difference at the 0.05 level (*p* < 0.05); the symbol “ns” indicates no significant differences between the two groups (*p* > 0.05). **(E)** Effects of different concentrations of QUE on porcine oocyte IVM. ANOVA of different superscript letter values in the same column is significantly different (*p* < 0.05). **(F)** Statistical analysis of the effects of different concentrations of QUE on porcine oocyte IVM. At least three separate experiments were performed, and the data are shown as the mean ± standard error of the mean (SEM). Scale bar = 200 μm. The symbol * indicates significant difference at the 0.05 level (*p* < 0.05).

The process of porcine COC *in vitro* maturation and aging is shown in Figure 1A. The COCs of the germinal vesicle (GV) stage were cultured in a mature medium supplemented with 0, 5, 10, and 20 µM for 42–44 h, and the COC expansion and first polar body extrusion were evaluated. We found that 5 µM QUE did not affect COC expansion, but 10 and 20 µM QUE significantly promoted COC expansion (Figures 1B–D). Furthermore, we found that 5 µM QUE significantly improved the PB1 extrusion rate of porcine oocytes, and 10 µM QUE improved the PB1 extrusion rate to a greater extent; however, 20 µM QUE had a lower effect on PB1 extrusion than 5 µM QUE **(** Figures 1E,F **)**.

### QUE exerts protective effects on the *in vitro* aging of oocytes

**FIGURE 2:**
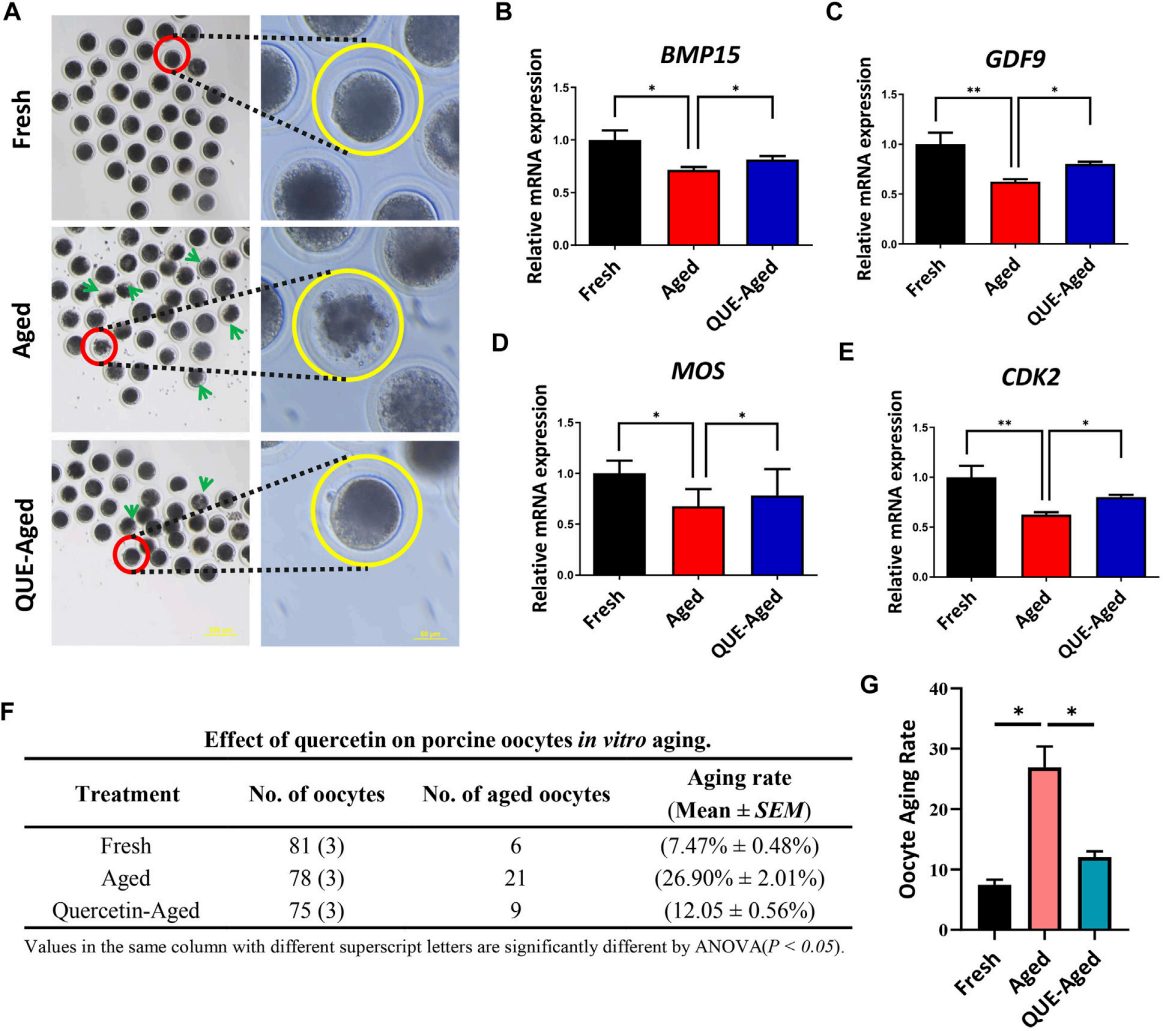
QUE supplementation inhibits the deterioration of the quality of the *in vitro* aged porcine oocytes. **(A)** Representative images of oocytes in fresh, aged, and QUE-aged groups. The abnormal cytoplasm of oocytes is marked with blue arrows. Scale bar = 200 μm in the left panel; Scale bar = 50 μm in the right panel. Expression levels of BMP15 **(B)**, GDF9 **(C)**, MOS **(D)** and CDK2 **(E)** in porcine oocytes of fresh, aged, and QUE-aged groups after 24 h of aging treatment. **(F)** QUE could protect oocytes from aging. ANOVA of different superscript letter values in the same column is significantly different (*p* < 0.05) (n = 234). **(G)** Statistical analysis of the effects of different concentrations of QUE on porcine oocytes in *in vitro* aging. The symbol * indicates significant difference at the 0.05 level (*p* < 0.05); and the symbol ** indicates significant difference at the 0.05 level (*p* < 0.01).

An excellent fresh oocyte presents a spherical structure, with a homogeneous and translucent cytoplasm surrounded by a uniform pellucid zone, whereas aged *in vitro* cultured oocytes tend to have a loosened and fragmented cytoplasm **(** Figure 2A). We found that 10 µM QUE significantly reduced oocyte cytoplasmic fragmentation (Figure 2A), thereby reducing the aging rate of *in vitro* cultured porcine oocytes (Figures 2F,G). Further analysis of the maturation-related promotion factors revealed that the expression levels of bone morphogenetic protein 15 (*BMP15*), growth differentiation factor 9 (*GDF9*), Moloney sarcoma oncogene (*MOS*), and cyclin-dependent kinase 2 (*CDK2*) were significantly downregulated in aged oocytes compared to those in fresh oocytes (Figures 2B–E). Supplementation with 10 µM QUE restored the expression of these maturation-related promoting factors (Figures 2B–E).

### QUE supplementation reduces the ROS levels in aged oocytes

**FIGURE 3:**
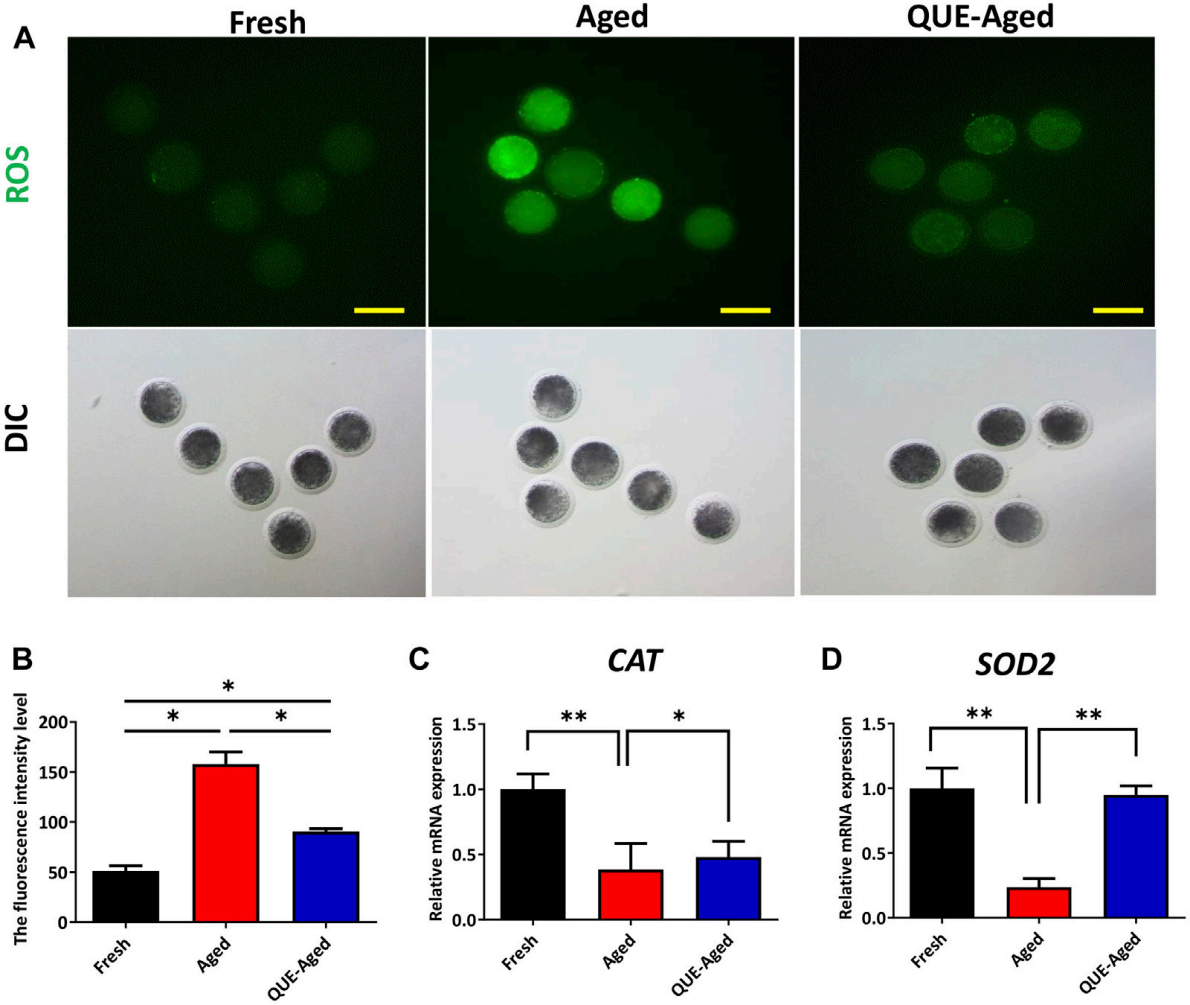
QUE reduces the oxidative stress in aged porcine oocytes. **(A)** Representative images of oocytes in fresh, aged, and QUE-aged groups stained with 2′, 7′-dichlorodihydrofluorescein diacetate (DCFH-DA) (n = 54). Scale bar = 200 μm. **(B)** Quantitative analysis of reactive oxygen species (ROS) levels in porcine oocytes of fresh, aged, and QUE-aged groups based on the intensity of the fluorescence signal. **(C)** Expression levels of catalase (*CAT*) in porcine oocytes of fresh, aged, and QUE-aged groups after 24 h of aging treatment. The symbol * indicates significant difference at the 0.05 level (*p* < 0.05), and the symbol ** indicates significant difference at the 0.05 level (*p* < 0.01). **(D)** Expression levels of superoxide dismutase 2 (*SOD2*) in porcine oocytes of fresh, aged, and QUE-aged groups after 24 h of aging treatment. The symbol * indicates significant difference at the 0.05 level (*p* < 0.05), and the symbol ** indicates significant difference at the 0.05 level (*p* < 0.01).

Oxidative stress derived from the accumulation of ROS during oocyte *in vitro* aging has been considered a key factor affecting oocyte quality. To examine whether QUE can reduce oxidative stress in *in vitro* aged oocytes, a DCFH-DA fluorescent probe was used to detect intracellular ROS levels in oocytes. The results showed that the fluorescence intensity of DCFH-DA was significantly higher in aged oocytes than in fresh oocytes (Figures 3A,B). QUE supplementation significantly reduced the ROS levels in aged oocytes, as reflected by the 43% decreased fluorescence intensity of DCFH-DA (Figures 3A,B). These results indicate that QUE could alleviate oxidative stress. Molecular analysis of the antioxidant enzymes showed that the expression levels of both *CAT* and *SOD2* decreased significantly in aged oocytes, and QUE supplementation increased the expression of both enzymes (Figures 3C,D). These findings indicate that QUE supplementation could restore the antioxidant capacity of aged oocytes.

### QUE supplementation restores the impaired mitochondrial function in aged oocytes

**FIGURE 4:**
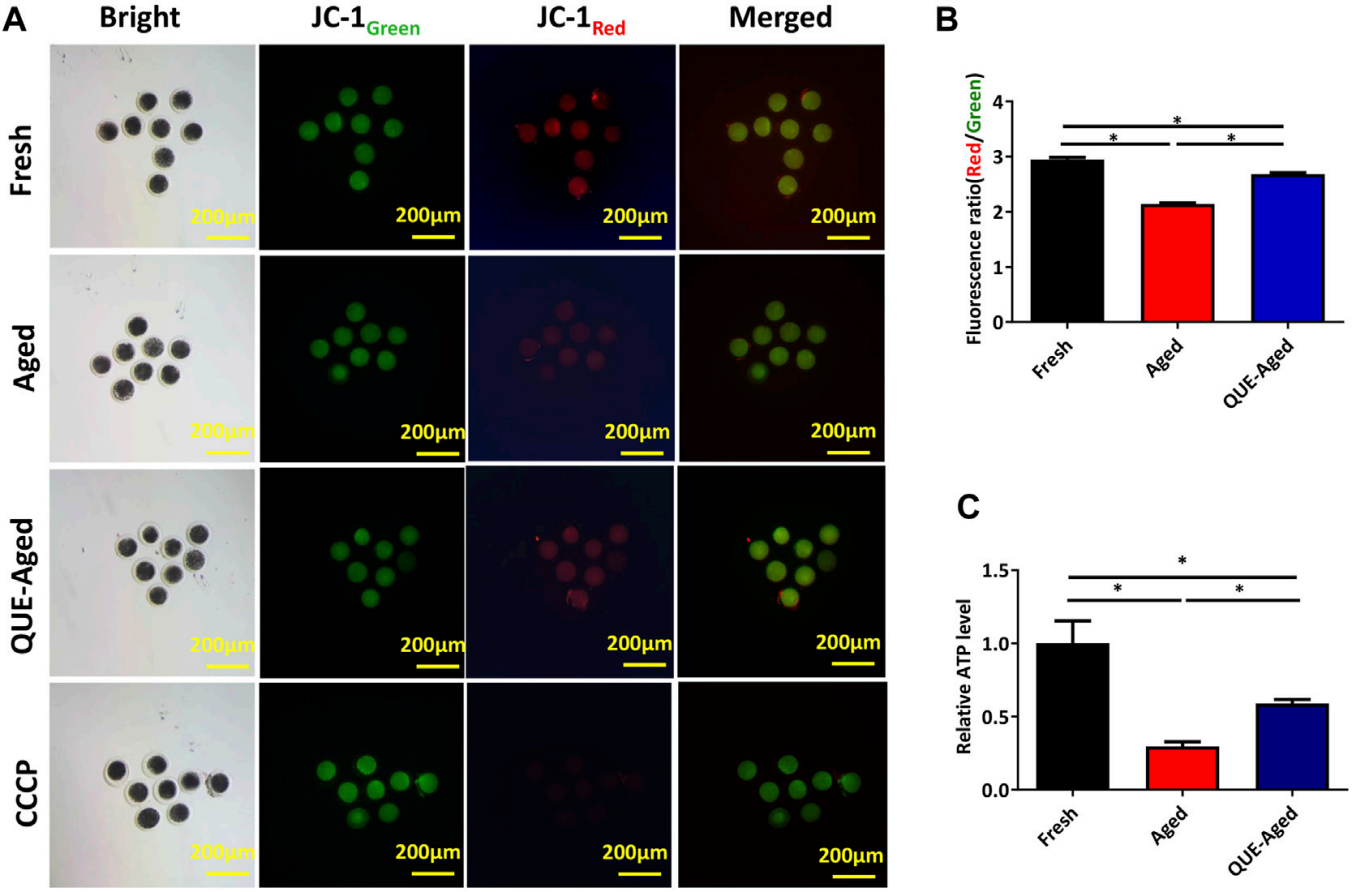
Effect of QUE on mitochondrial function in aged porcine oocytes. **(A)** Representative images of JC-1 staining of porcine oocytes of fresh, aged, and QUE-aged groups. Scale bar = 200 μm. **(B)** JC-1_Red/Green_ fluorescence signal was quantitatively analyzed in porcine oocytes of fresh, aged, and QUE-aged groups. The symbol * indicates significant difference at the 0.05 level (*p* < 0.05). **(C)** ATP content was quantitatively analyzed in fresh, aged, and QUE-aged groups. The symbol * indicates significant difference at the 0.05 level (*p* < 0.05).

ROS accumulation can induce mitochondrial dysfunction. Mitochondrial function can be indicated by the mitochondrial membrane potential (ΔΨm), which is critical for maintaining the physiological function of the respiratory chain to generate ATP. Therefore, we used the JC-1 probe to determine whether the mitochondrial function was impaired in aged oocytes. The significantly reduced red/green ratio compared with that in fresh oocytes (Figures 4A,B) implied impaired mitochondrial function in aged oocytes. QUE supplementation efficiently restored the impaired mitochondrial function in aged oocytes, as reflected by a 25% increased red/green ratio (Figures 4A,B), indicating that impaired mitochondrial function was restored. Further detection of significantly reduced ATP content in aged oocytes confirmed impaired mitochondrial function (Figure 4C), while supplementation with QUE increased 40% ATP production in aged oocytes (Figure 4C), indicating that impaired mitochondrial function was restored.

### QUE supplementation reduces the early apoptosis in aged oocytes

**FIGURE 5:**
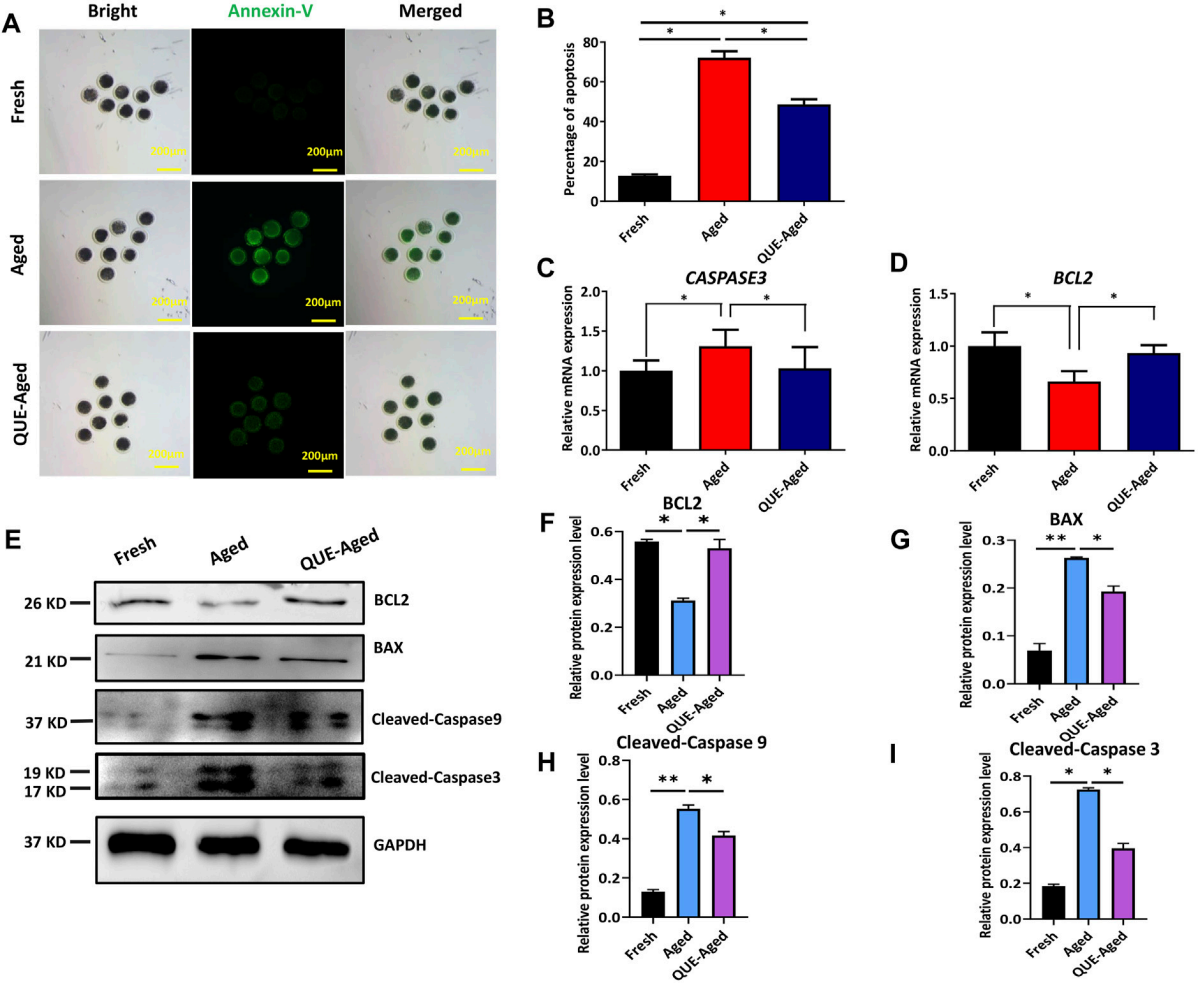
QUE inhibits the early apoptosis of aged porcine oocytes. **(A)** Representative images of annexin-V staining of porcine oocytes of fresh, aged, and QUE-aged groups. Scale bar = 200 μm. **(B)** Analysis of the ratio in porcine oocytes of fresh, aged, and QUE-aged groups (n = 470). The symbol * indicates significant difference at the 0.05 level (*p* < 0.05). **(C)** Expression levels of *CASPASE3* in porcine oocytes of fresh, aged, and QUE-aged groups after 24 h of aging treatment. The symbol * indicates significant difference at the 0.05 level (*p* < 0.05). **(D)** Expression levels of B-cell lymphoma-2 (*BCL2*) in porcine oocytes in fresh, aged, and QUE-aged groups after 24 h of aging treatment. The symbol * indicates significant difference at the 0.05 level (*p* < 0.05). **(E)** Western blotting analysis of BCL2, Bcl-2-associated X (BAX), cleaved caspase 3, and cleaved caspase 9 protein levels in fresh, aged, and QUE-aged groups (n = 600). **(F)** Expression levels of BCL2 in porcine oocytes of fresh, aged, and QUE-aged groups. The symbol * indicates significant difference at the 0.05 level (*p* < 0.05). **(G)** Expression levels of BAX in porcine oocytes of fresh, aged, and QUE-aged groups. The symbol * indicates significant difference at the 0.05 level (*p* < 0.05). **(H)** Expression levels of cleaved caspase 9 in porcine oocytes of fresh, aged, and QUE-aged groups. The symbol * indicates significant difference at the 0.05 level (*p* < 0.05). **(I)** Expression levels of cleaved caspase 3 in porcine oocytes of fresh, aged, and QUE-aged groups. The symbol * indicates significant difference at the 0.05 level (*p* < 0.05).

Oxidative stress can induce early apoptosis during post-ovulatory oocyte aging (Lord and Aitken 2013). Early apoptotic oocytes express phosphatidylserines on the outer leaflet of the plasma membrane, which can be stained with fluorophore-labeled annexin-V (Guemra et al.). We found that fresh oocytes could not be stained by annexin-V (Figure 5A), but aged oocytes were strongly stained by annexin-V (Figure 5A). QUE supplementation reduced 23% annexin-V staining in aged oocytes (Figures 5A,B). We further detected the mRNA expression levels of the pro-apoptotic gene, *CASPASE3*, and the anti-apoptotic gene, B-cell lymphoma-2 (*BCL2*), using RT-qPCR. The results demonstrated that *CASPASE3* was significantly upregulated in aged oocytes and QUE supplementation reduced *CASPASE3* expression in aged oocytes (Figure 5C), which was confirmed by Western blot analysis of the expression of cleaved caspase 3 protein and cleaved caspase 9 protein (Figures 5E,H,I). In contrast, the expression of *BCL2* was significantly downregulated in aged oocytes, and QUE supplementation upregulated *BCL2* expression in aged oocytes (Figure 5D), which was confirmed by Western blot analysis of BCL2 protein expression (Figures 5E,F). Additionally, Western blotting analysis of the pro-apoptotic factor, Bcl-2-associated X (BAX), showed that it was significantly upregulated in aged oocytes, and QUE supplementation downregulated BAX expression to a certain extent in aged oocytes (Figures 5E,G). These results suggest that QUE supplementation inhibits early apoptosis during oocyte *in vitro* aging.

### QUE supplementation inhibits the autophagy of *in vitro* aged oocytes

**FIGURE 6:**
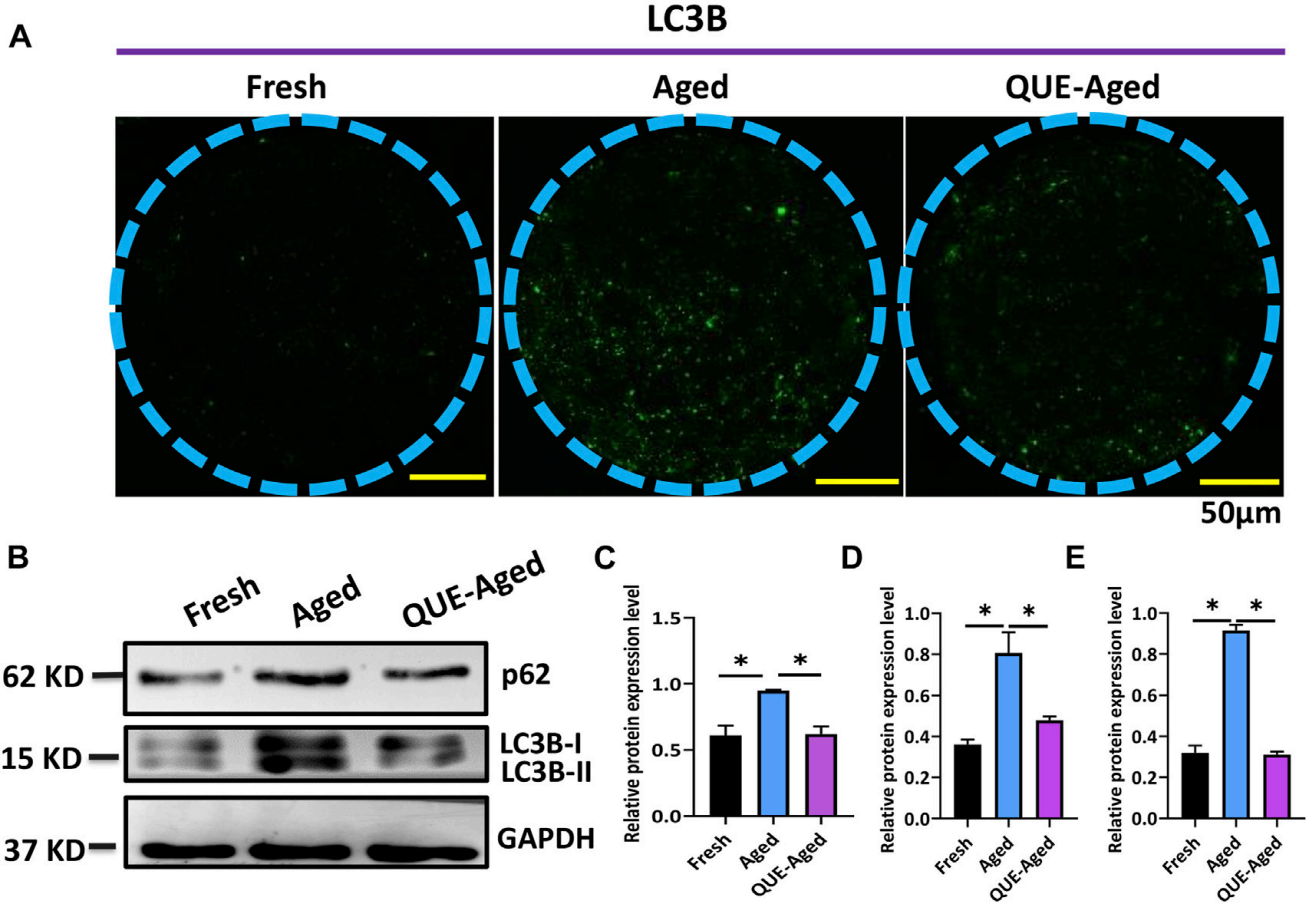
QUE attenuates the autophagic activity in aged porcine oocytes. **(A)** Representative images of fresh, aged, and QUE-aged groups stained with LC3B. Scale bar = 50 μm. **(B)** Western blotting analysis of LC3B and p62 protein levels in fresh, aged, QUE-aged groups (n = 600). **(C)** Expression levels of p62 in porcine oocytes of fresh, aged, and QUE-aged groups. The symbol * indicates significant difference at the 0.05 level (*p* < 0.05). **(D)** Expression levels of LC3B-I in porcine oocytes of fresh, aged, and QUE-aged groups. The symbol * indicates significant difference at the 0.05 level (*p* < 0.05). **(E)** Expression levels of LC3B-II in porcine oocytes of fresh, aged, and QUE-aged groups (n = 200). The symbol * indicates significant difference at the 0.05 level (*p* < 0.05).

A complex interplay between oxidative stress and autophagy has been found in the context of aged oocytes (Wang et al., 2021). Therefore, we investigated whether QUE affects autophagy in porcine oocytes *in vitro*. Through immunofluorescent staining of the autophagic marker LC3B, we found few positive staining dots in fresh oocytes but universally distributed positive staining dots in aged oocytes (Figure 6A), indicating that autophagy had occurred in aged oocytes. QUE supplementation reduced the quantity of LC3B positive dots (Figure 6A), implying that QUE can attenuate autophagic activity in aged porcine oocytes. This was confirmed by Western blotting analysis of the autophagic activity reporters p62 and LC3B (LC3B-I and LC3B-II), where QUE supplementation attenuated the upregulation of these proteins (Figures 6B–E).

### QUE supplementation reduces the spindle abnormalities in aged oocytes

**FIGURE 7:**
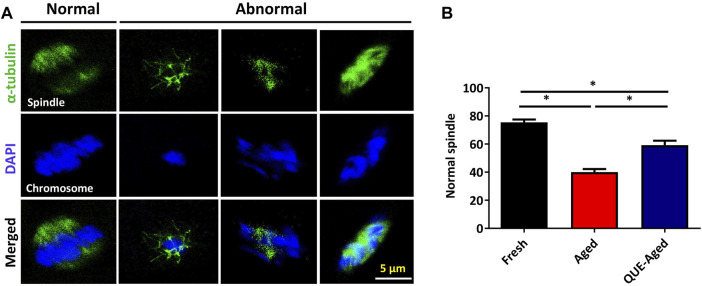
QUE reduces the spindle abnormalities in aged porcine oocytes. **(A)** Representative immunofluorescent figures of normal and abnormal spindle morphologies and chromosome alignment in fresh, aged, and QUE-aged groups. Scale bar = 5 μm. **(B)** Percentage of the normal spindle in porcine oocytes of fresh, aged, and QUE-aged groups. The symbol * indicates significant difference at the 0.05 level (*p* < 0.05).

Accumulated ROS levels cause cytoskeletal defects that may lead to spindle abnormalities in aged oocytes (Ren et al., 2012). Therefore, we further examined whether QUE could protect *in vitro* aged oocytes from spindle organization and chromosome alignment abnormalities. Spindle morphology and chromosome alignment were analyzed (Figure 7A). We found that the percentage of normal spindle/chromosome structures decreased significantly in aged oocytes, and QUE supplementation increased the 19% normal spindle in aged oocytes (Figure 7B). This result suggests that QUE supplementation can reduce spindle/chromosomal abnormalities in *in vitro* aged porcine oocytes.

## Discussion

Numerous studies based on cellular and mouse models have shown that QUE has therapeutic effects on metabolic disorders through a variety of mechanisms, such as increasing adiponectin, decreasing leptin, and antioxidant activity (Mariani et al., 2008). The *in vitro* maturation and aging of porcine oocytes involve complex metabolic activity, and whether QUE can improve the IVM rate of porcine oocytes and protect them from aging has not yet been investigated. In the current study, we demonstrated that 10 µM QUE could efficiently improve porcine oocyte IVM (Figure 1), which is consistent with the findings of previous studies (Orlovschi et al., 2014). Subsequently, we found that QUE improved the quality of aged porcine oocytes by reducing the percentage of oocytes with loosening and fragmented cytoplasm (Figure 2) and restored the impaired expression of maturation-promoting factors (MPFs), including *BMP15*, *GDF9*, *MOS*, and *CDK2* (Figure 2). BMP15 and GDF9 are members of the transforming growth factor-β (TGF-β) family, and their roles in promoting oocyte maturation and cumulus expansion have been well-confirmed in many species, including mice, sheep, dogs, pigs, and humans (Galloway et al., 2000; Di Pasquale, Beck-Peccoz, and Persani 2004). The expression of *MOS* in mammalian species is initiated immediately after oocyte germinal vesicle breakdown, playing a critical role in the oocyte maturation process *via* the activation and/or stabilization of MPF (Paronetto et al., 2004). CDK2 kinase, a G1/S regulator, is activated during meiotic maturation of oocytes, similar to *MOS*, and participates in metaphase II arrest in mature oocytes (Ortega et al., 2003). Therefore, restoration of the impaired expression of *BMP15*, *GDF9*, *MOS*, and *CDK2* in aged porcine oocytes would improve their maturation quality.

Deterioration of the quality of *in vitro* aged oocytes has been associated with increased oxidative stress, mainly due to excessive accumulation of ROS (Prozorovski et al., 2008; Caito et al., 2010). We found that the ability of QUE to protect aged porcine oocytes from further deterioration of quality is mainly due to its antioxidant activity. This was evidenced by the reduction in ROS levels and the restoration of impaired *CAT* and *SOD2* expression (Figure 3). Increased expression of *CAT* and *SOD2* enhances the antioxidant ability of oocytes (Niu et al., 2020). It is well known that excessive accumulation of ROS can impair mitochondrial function (Chen et al., 2003; Khazaei and Aghaz 2017). We believe that the antioxidant activity of QUE can effectively alleviate impaired mitochondrial function in aged porcine oocytes, as reflected by the partial restoration of mitochondrial membrane potential (ΔΨm) and ATP production (Figure 4). Mitochondrial dysfunction may activate early apoptosis (Lord and Aitken 2013), and the restoration of mitochondrial function in aged porcine oocytes by QUE also attenuates early apoptosis in oocytes (Figure 5). In addition, mitochondrial dysfunction activates cellular autophagy (Woods, Khrapko, and Tilly 2018). We also found that the restoration of mitochondrial function in aged porcine oocytes by QUE inhibited autophagic activity, as reflected by the reduced expression of LC3B and p62 protein in aged oocytes (Figure 6), which is in accordance with previous studies showing that QUE can moderate the internal environment and inhibit autophagy in aging oocytes by activating the PI3K/Akt signaling pathway (Wu et al., 2017). In addition to impairing mitochondrial function, excessive oxidative stress has also been shown to affect spindle assembly and chromosome alignment in aging mouse and pig oocytes (Ma et al., 2015). We found that attenuation of oxidative stress by QUE could effectively reduce spindle abnormalities in aged oocytes (Figure 7), inhibiting further deterioration of oocyte quality.

## Conclusion

**FIGURE 8:**
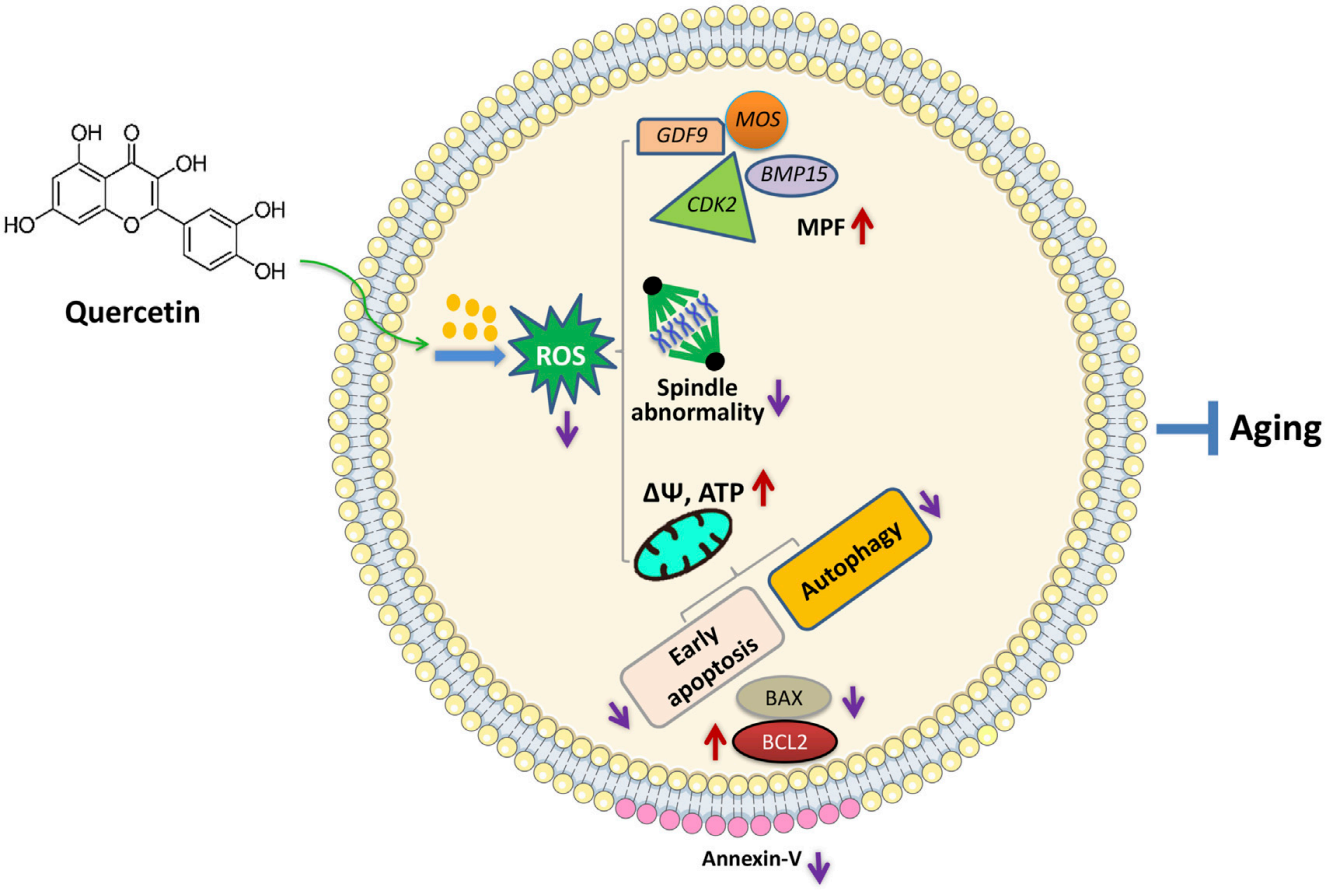
Proposed regulatory effect of QUE on porcine oocytes in *in vitro* aging. Supplementation of QUE reduces the intracellular ROS levels in aged porcine oocytes, which leads to restoration of impaired mitochondrial functions and reduced spindle abnormalities. The restored mitochondrial functions indicated by increased mitochondrial membrane potential (ΔΨm) and ATP content can further inhibit autophagic activity and early apoptosis reflected by the increased expression of BCL2, reduced expression of BAX, and reduced annexin-V staining. In addition, QUE restores the expression levels of maturation-promoting factors (MPFs) (*BMP15*, *GDF9*, *MOS*, and *CDK2*). Therefore, QUE protects porcine oocytes from *in vitro* aging.

In conclusion, we found that QUE supplementation can effectively improve the IVM of porcine oocytes and the quality of *in vitro* aged porcine oocytes, mainly through its antioxidant effects, which can reduce ROS accumulation, resulting in the restoration of impaired mitochondrial functions and prevention of early apoptosis, autophagy, and spindle abnormalities (Figure 8). This can aid in the development of new strategies for improving the IVM of porcine oocytes and provide insight into the factors regulating the porcine oocyte aging process.

## Data Availability

The original contributions presented in the study are included in the article/supplementary material, further inquiries can be directed to the corresponding author/s.
