# Supplementary material for: Quercetin protects porcine oocytes from in vitro aging by reducing oxidative stress and maintaining the mitochondrial functions
Source: Front Cell Dev Biol. 2022 Oct 5;10:915898. doi: 10.3389/fcell.2022.915898 (PMC9581393; doi:10.3389/fcell.2022.915898)
Supplement: Supplementary file 2 [file Table1.DOCX]

**Table S1.** Primer sequences used for RT-qPCR analysis.

| Name | sequence（5'-3'） | Length (bp) | Annealing℃ | Reference/accession numbers |
| --- | --- | --- | --- | --- |
| *GAPDH*-F | GGGCGTGAACCATGAGAAGT | 230 | 60 | NM_001206359.1 |
| *GAPDH*-R | AAGCAGGGATGATGTTCTGG |  |  |  |
| *BMP15*-F | CCCTCGGGTACTACACTATG | 192 | 60 | AF_458070.2 |
| *BMP15*-R | GGCTGGGCAATCATATCC |  |  |  |
| *GDF9*-F | GAGCTCAGGACACTGTAAGCT | 272 | 60 | NM_001001909.1 |
| *GDF9*-R | CTTCTCGTGGATGATGTTCTG |  |  |  |
| *MOS*-F | TGGGAAGAAACTGGAGGACA | 121 | 60 | NM_001113219.1 |
| *MOS*-R | TTCGGGTCAGCCCAGTTCA |  |  |  |
| *CDK2*-F | AAACAAGTTGACGGGAGA | 298 | 60 | XM_001928965 |
| *CDK2*-R | GTGAGAATGGCAGAAAGC |  |  |  |
| *CAT*-F | ACACACCTGAAGGATCCGGA | 150 | 60 | NM_214301.2 |
| *CAT*-R | AACCAGCTTGAAAGTGTGCG |  |  |  |
| *SOD2*-F | TCAAGAGAGGCACGTTGGAG | 157 | 60 | NM_001190422.1 |
| *SOD2*-R | TCTGCCCAAGTCATCTGGTT |  |  |  |
| *CASPASE3-*F | TTTGCGTGCTTCTAAGCCAT | 147 | 60 | NM_214131.1 |
| *CASPASE3-*R | GGCAGGCCTGAATTATGAAA |  |  |  |
| *BCL2-*F | GAACTGGGGGAGGATTGTGG | 164 | 60 | XM_003121700.3 |
| *BCL2-*R | CATCCCAGCCTCCGTTATCC |  |  |  |

**F, forward; R, reverse.**

**Table S2.** List of antibodies used in this study.

| Antibody | Catalog Code | Source | Host | Dilution | |
| --- | --- | --- | --- | --- | --- |
|  |  |  |  | IF | WB |
| LC3B-1 | ab48394 | Abcam | Rabbit | 1:200 |  |
| LC3B-2 | 3868S | Cell Signaling Technology | Rabbit |  | 1:1000 |
| α-Tubulin | ab64503 | Abcam | Rabbit | 1:200 |  |
| p62 | Ab109012 | Abcam | Rabbit |  | 1:1000 |
| Cleaved Caspase3 | 9664S | Cell Signaling Technology | Rabbit |  | 1:1000 |
| Cleaved Caspase9 | AF5240 | Affinity | Rabbit |  | 1:1000 |
| BAX | T40051S | Abmart | Rabbit |  | 1:1000 |
| BCL2 | T40056S | Abmart | Rabbit |  | 1:1000 |
| Goat Anti-Rabbit IgG | Ab6721 | Abcam | Goat |  | 1:5000 |
| GAPDH | ab9485 | Abcam | Rabbit |  | 1:1000 |
