# Supplementary figures and images for: Quercetin protects porcine oocytes from in vitro aging by reducing oxidative stress and maintaining the mitochondrial functions
Source: Front Cell Dev Biol. 2022 Oct 5;10:915898. doi: 10.3389/fcell.2022.915898 (PMC9581393; doi:10.3389/fcell.2022.915898)

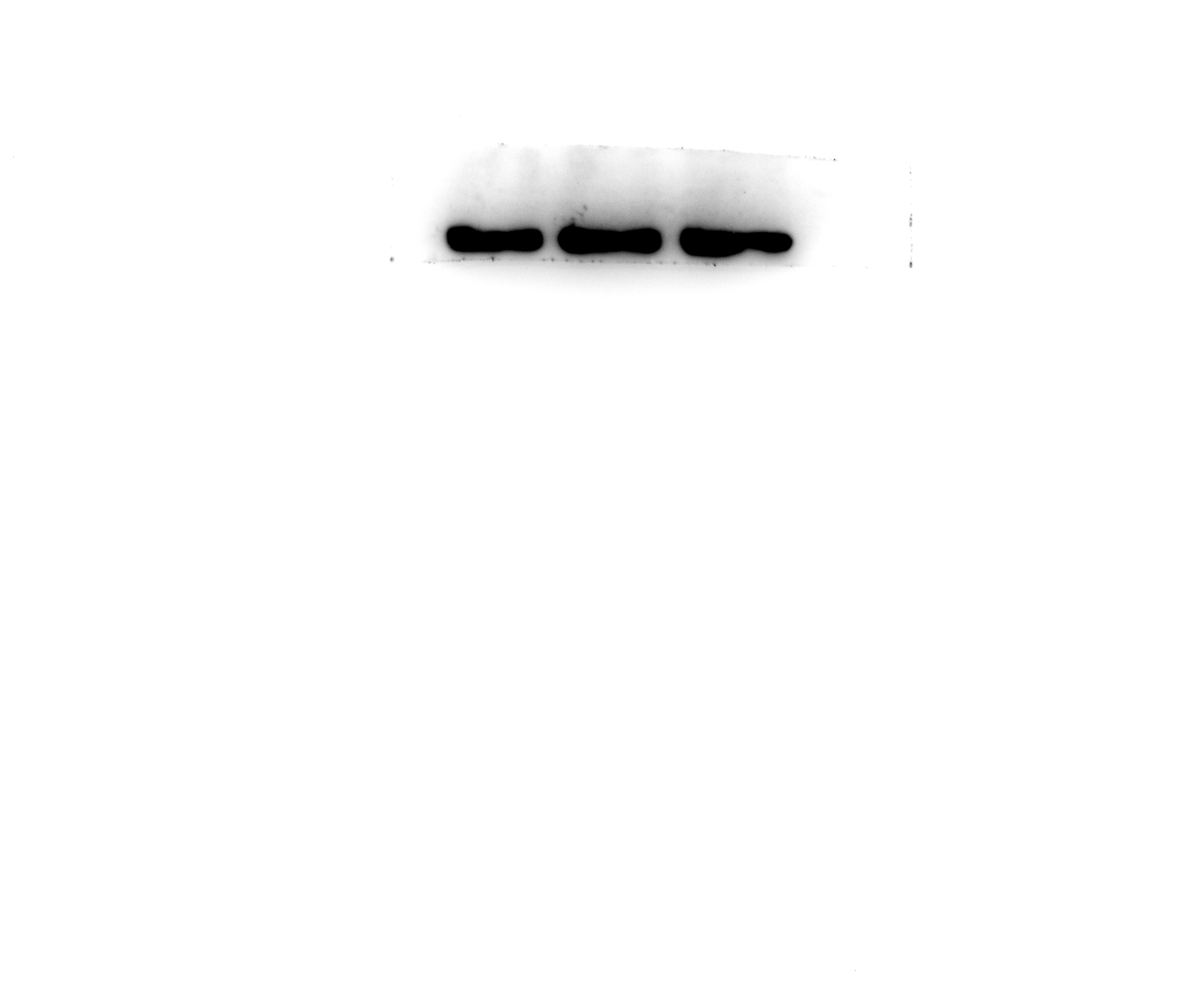

Supplement: Supplementary file 3 [file Image2.TIF]

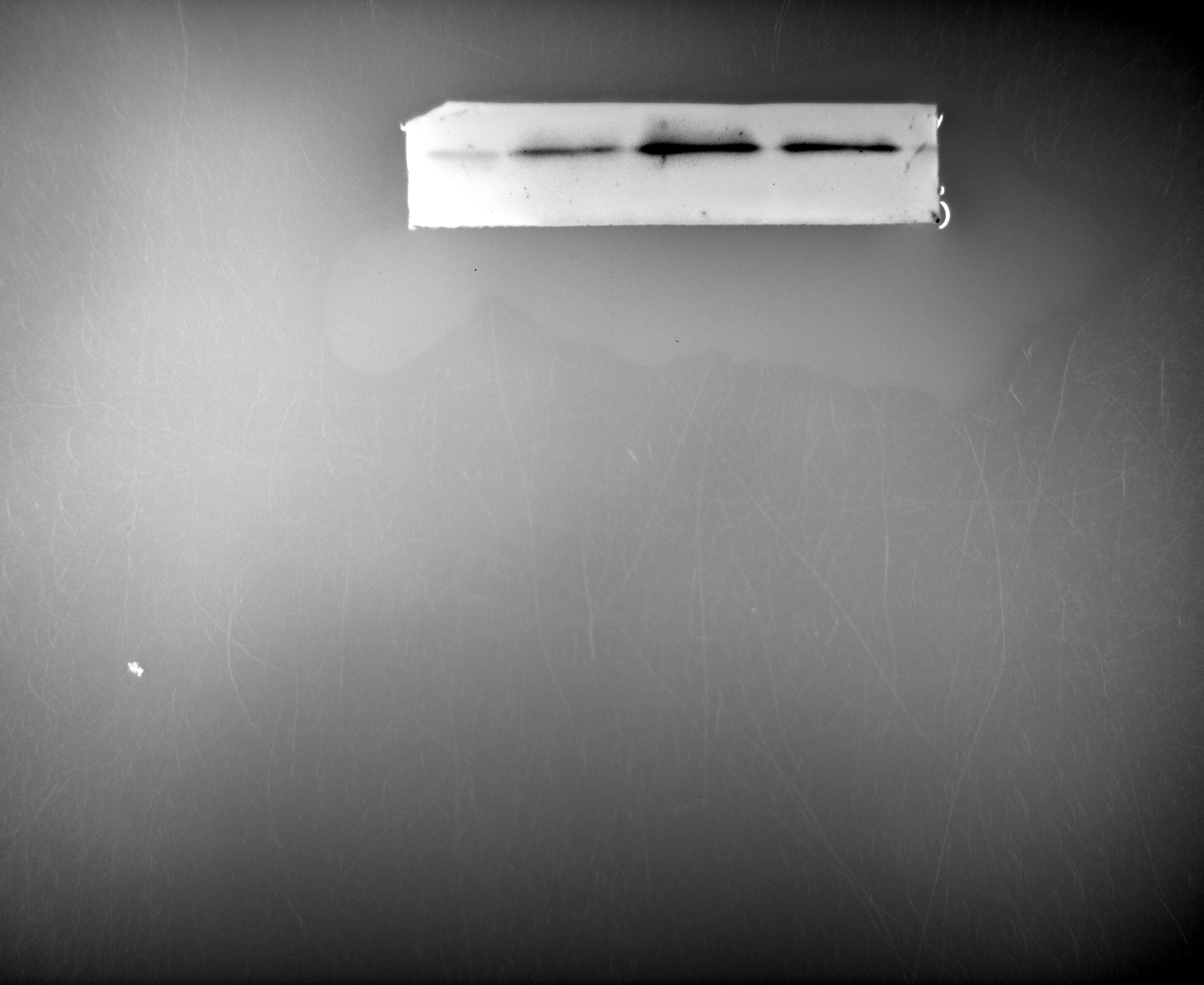

Supplement: Supplementary file 4 [file Image1.TIF]
