# Supplementary material for: Quercetin protects porcine oocytes from in vitro aging by reducing oxidative stress and maintaining the mitochondrial functions
Source: Front Cell Dev Biol. 2022 Oct 5;10:915898. doi: 10.3389/fcell.2022.915898 (PMC9581393; doi:10.3389/fcell.2022.915898)

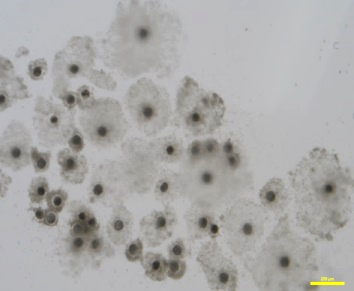

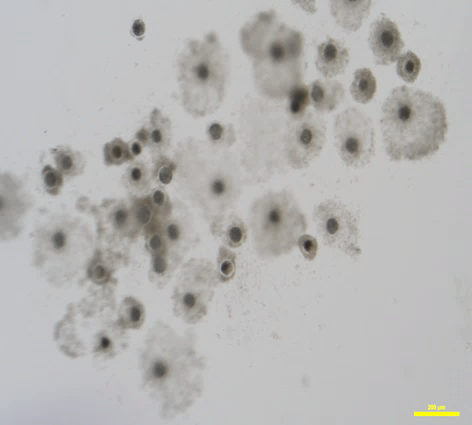

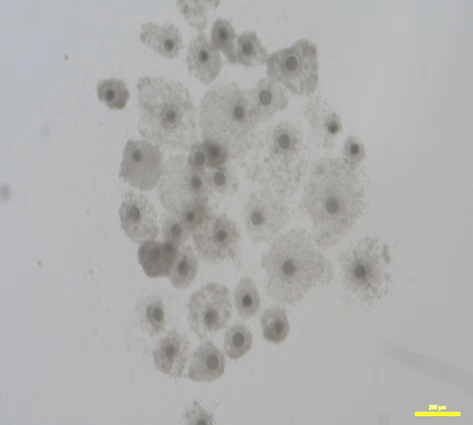

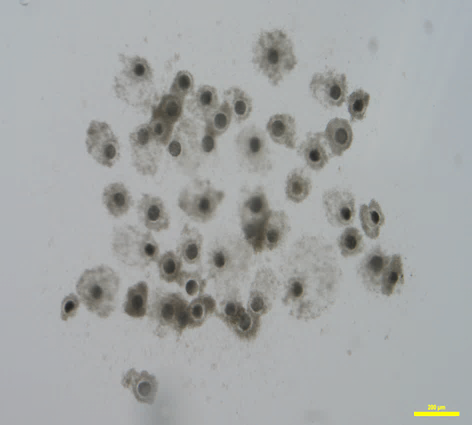


10 μM

0 μM

5 μM


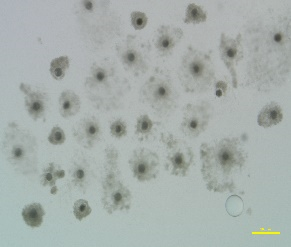


20 μM

**Effects of quercetin at different concentrations on porcine oocytes IVM**

| **Concentration (µM)** | **Total oocytes(repeat number)** | **Number of oocytes with first polar body** | **Rate of PB1(mean±*SEM*)** |
| --- | --- | --- | --- |
| **0** | **160(3)** | **114** | **(71.25% ± 0.47%)^a^** |
| **5** | **142(3)** | **108** | **(76.06% ± 1.15%)^b^** |
| **10** | **184(3)** | **154** | **(83.70% ± 0.64%)^c^** |
| **20** | **176(3)** | **131** | **(74.43%± 1.61%)^ab^** |

**Note: Values in the same column with different superscript letters differ significantly by ANOVA (*P < 0.05*).**

**Effect of** **q****uercetin on cumulus expansion of porcine cumulus oocyte complex IVM**

| **Concentration (μM)** | **No. of oocytes** | **Grade 1**  **(1 scores)** | **Grade 2**  **(2 scores)** | **Grade 3**  **(3 scores)** | **Mean ± *SEM*** |
| --- | --- | --- | --- | --- | --- |
| **0** | **112(3)** | **11** | **26** | **75** | **(2.57±0.04)^a^** |
| **5** | **105(3)** | **10** | **23** | **72** | **(2.58±0.04)^a^** |
| **10** | **124(3)** | **11** | **16** | **97** | **(2.69±0.01)^b^** |
| **20** | **108(3)** | **11** | **14** | **83** | **(2.67±0.01)^b^** |

**Note: Values in the same column with different superscript letters differ significantly by ANOVA (*P* < 0.05).**

Fresh


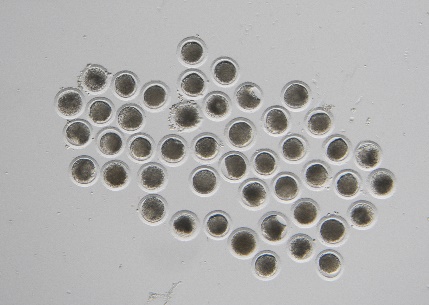

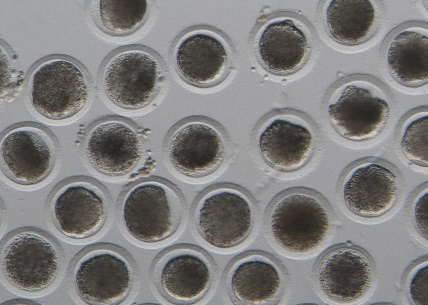


Aged


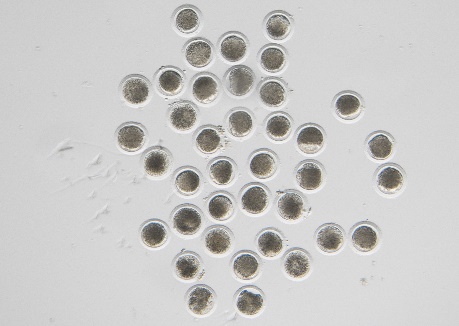

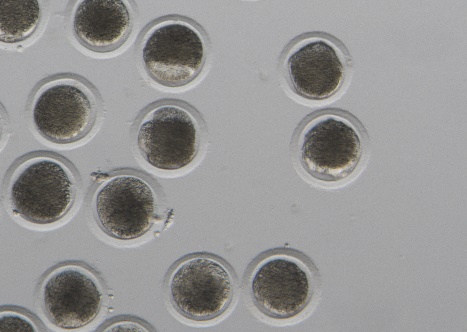


Aged + QUE


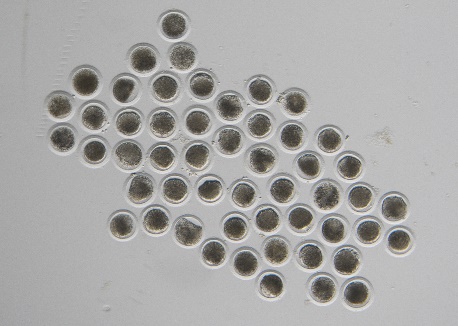

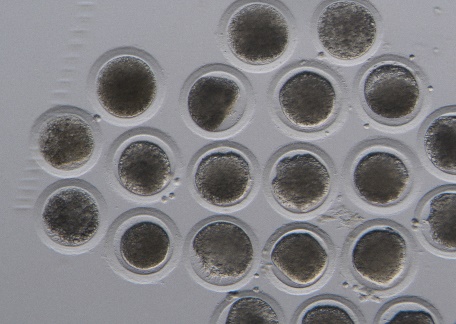


**Quercetin played a protective role on oocytes *in vitro* Aging**

| **Treatment** | **No. of oocytes** | **No. of aged oocytes** | **Aging rate**  (**Mean ± *SEM***) |
| --- | --- | --- | --- |
| **Fresh** | **81(3)** | **6** | **(7.47% ± 0.48%)** |
| **Aged** | **78(3)** | **21** | **(26.90% ± 2.01%)** |
| **Quercetin -Aged** | **75(3)** | **9** | **(12.05 ± 0.56%)** |

**Note: Values in the same column with different superscript letters differ significantly by ANOVA (*P < 0.05*).**

Fresh


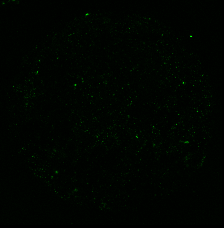


Aged


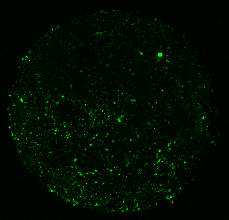


Aged-QUE


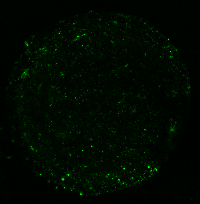


**QUE played a protective role on oocytes *in vitro* Aging**

| **Treatment** | **No. of oocytes** | **No. of aged oocytes** | **Aging rate**  (**Mean ± *SEM***) |
| --- | --- | --- | --- |
| **Fresh** | **81(3)** | **6** | **(7.47% ± 0.48%)** |
| **Aged** | **78(3)** | **21** | **(26.90% ± 2.01%)** |
| **QUE -Aged** | **75(3)** | **9** | **(12.05 ± 0.56%)** |

**Note: Values in the same column with different superscript letters differ significantly by ANOVA (*P < 0.05*).**

**Normal**


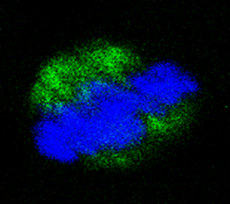

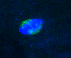

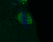


**Abnormal**


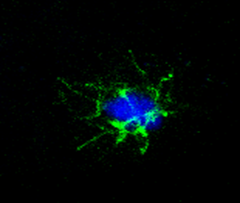

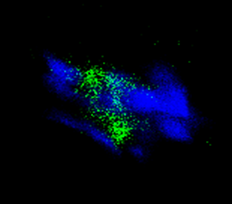

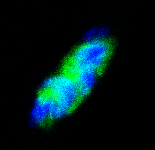

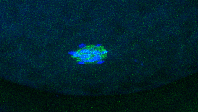


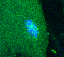

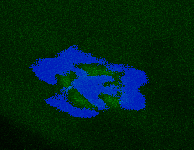

Supplement: Supplementary file 5 [file Table2.DOCX]
